# High hepatic macrophage activation and low liver function in stable Wilson patients - a Danish cross-sectional study

**DOI:** 10.1186/s13023-018-0910-7

**Published:** 2018-09-21

**Authors:** Jessica Björklund, Tea Lund Laursen, Thomas Damgaard Sandahl, Holger Jon Møller, Hendrik Vilstrup, Peter Ott, Henning Grønbæk

**Affiliations:** 1 Department of Hepatology and Gastroenterology, Aarhus University Hospital, DK-8000 Aarhus, Denmark; 2 Department of Clinical Biochemistry, Aarhus University Hospital, Aarhus, Denmark

**Keywords:** Wilson disease, Macrophages, Liver, Metabolic liver function, sCD163

## Abstract

**Background:**

Hepatic macrophage (Kupffer cell) hyperplasia is often described in Wilson’s disease (WD). In many liver diseases, Kupffer cell activation is related to disease severity, liver function, and fibrosis but the importance in WD is unknown. Kupffer cell activation can be assessed by the P-concentration of soluble (s)CD163, metabolic liver function by the galactose elimination capacity (GEC), and fibrosis by Fibroscan. We investigated the associations between sCD163, selected inflammatory cytokines, GEC, and liver fibrosis in Danish WD patients.

**Methods:**

In a cross-sectional design, we studied 29 stable and well-treated patients (male/female15/14) with a median age of 35 years (IQR 24–50). P-sCD163 and cytokines were measured by ELISA. The GEC was measured by intra-venous galactose loading.

**Results:**

The median P-sCD163 value at 2.96 mg/L (1.97–3.93) was high in the normal range (0.7–3.9) and seven patients (24%) had a value above the upper normal value. sCD163 correlated with TNF-α, IL-6 and IL-8 (rho> 0.50, *p* < 0.005). A higher sCD163 value was closely associated with a lower GEC (rho = − 0.51, *p* = 0.02). sCD163 was not related to the liver fibrosis indices.

**Conclusions:**

Stable WD patients showed various degrees of Kupffer cell activation which was accompanied by loss of metabolic liver function. Neither activation nor liver function was related to liver fibrosis. The findings suggest that in WD inflammatory Kupffer cell activation may be involved in the loss of liver function over time. sCD163 may serve as a non-invasive biomarker of loss of liver function in WD, which the degree of fibrosis evidently may not.

This study is registered at clinical trials with name: “sCD163 and sMR in Wilsons Disease - Associations With Disease Severity and Fibrosis”, NCT02702765. Date of registration: 26.02.16. Date of enrolment of the first participant to the trial: 17.03.16. ULR: https://clinicaltrials.gov/ct2/show/NCT02702765.

## Background

Wilson’s disease (WD) is a rare autosomal recessive disease, in Europe mostly caused by mutations in the ATP7B gene which encodes a copper regulating protein leading to a defect in biliary copper excretion [1]. Copper accumulates in the liver and the central nervous system, cornea, kidney, joints, and cardiac muscle with impaired function of the affected organs [2]. WD usually presents with hepatic or neuropsychiatric symptoms that vary markedly among patients [3].

The histological features suggest that copper induced immune activation is likely to contribute to the liver damage [4], but the mechanism is very scarcely studied. However, with progression of the disease, the numbers of resident macrophages (Kupffer cells) increase, along with pericellular fibrosis [4]. This suggests that inflammatory activation of Kupffer cells may play a role in the toxico-immuno-genesis of the liver damage in WD, as is known to be the case in alcoholic and non-alcoholic cirrhosis [5, 6]. The degree of Kupffer cell activation can be assessed by the P-concentration of soluble (s)CD163. CD163 is a lineage specific marker of monocyte/macrophage activation. CD163 functions as a hemoglobin-haptoglobin scavenger receptor and is shed from macrophages in the liver upon activation [7–9]. Increased sCD163 is associated with disease severity in several liver diseases [10–12] and with prognosis in patients with liver cirrhosis and acute-on-chronic liver failure [13].

Our aim was to explore if WD patients exhibit Kupffer cell activation and whether this might have a hepatic functional correlate. We, therefore, measured P-sCD163, the galactose elimination capacity (GEC) as a test of metabolic liver function, and the degree of fibrosis by transient elastography in a cohort of stable patients [14, 15].

## Methods

### Study design and population

**Fig. 1:**
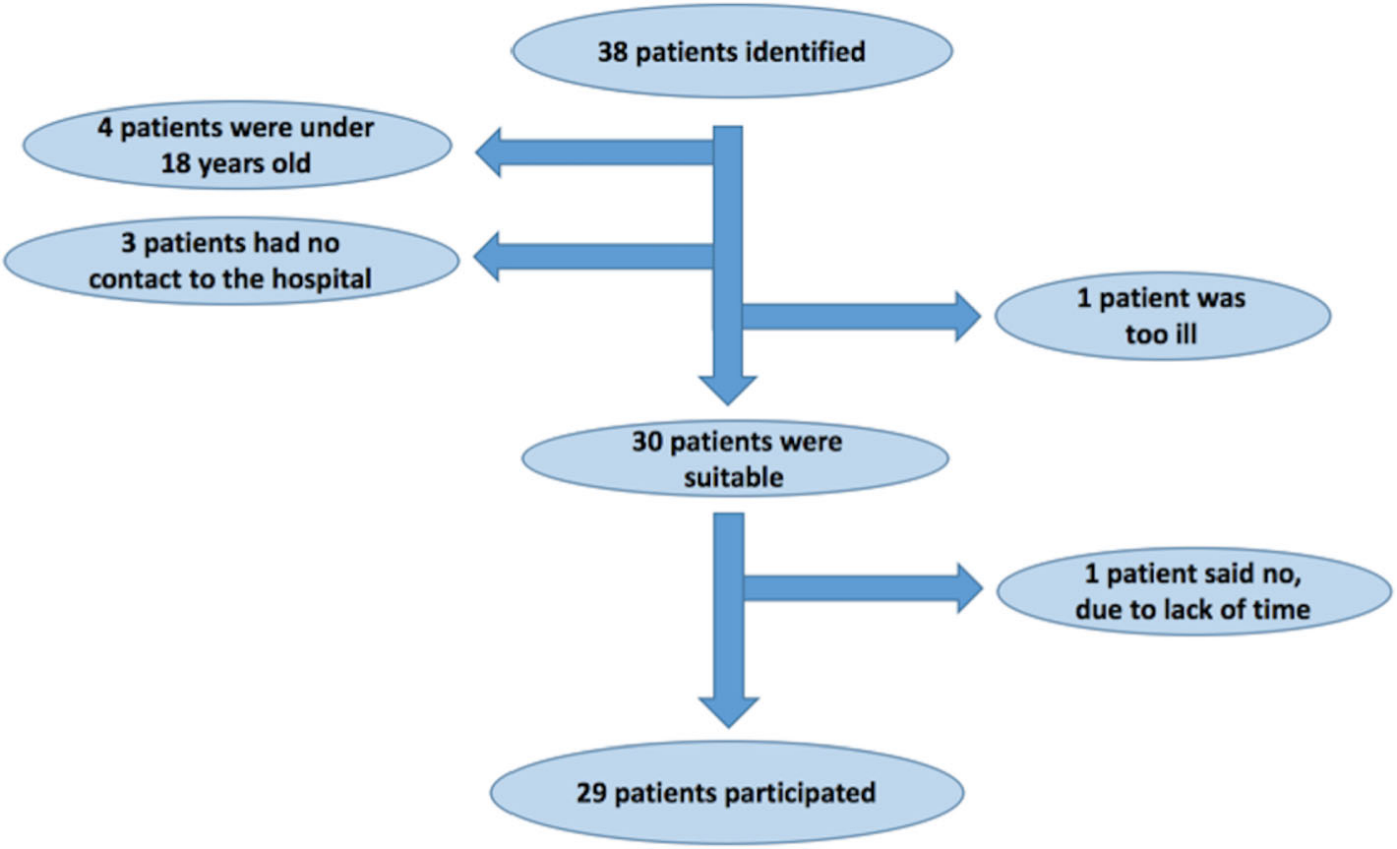
Patient flowchart

We studied WD patients attending our Danish Wilson Center during the inclusion period February – November 2016. Inclusion criteria were age above 18 years and an established diagnosis of WD. The only exclusion criteria were inability to collect urine or liver transplantation prior to inclusion. We identified 38 non-transplanted patients. Four were below 18 years, 3 had no contact to the hospital, and one was too ill to participate (Fig. 1). Thus, a total of 29 patients were included. The control group was 19 healthy volunteer persons previously described [5, 16]. All participants signed informed consent forms.

The study was approved by The Central Denmark Region Committees on Health Research Ethics (50611), registered at clinicaltrials.gov (NCT02702765), and approved by the Danish Data Protection Agency (1–16–02-614-15). The study was conducted in accordance with The Helsinki Declaration.

### Blood samples and biochemical analyses

Blood samples drawn at admission included liver enzymes, coagulation factors, hematology, fluid balance, kidney function measures, and serum zinc. We used standard methods at the local Department of Clinical Biochemistry. We calculated the MELD score to show patients characteristics for all WD patients even in the absence of end stage liver disease. The cytokines tumor necrosis factor-α (TNF-α), interleukin (IL)-6, and IL-8 were analyzed in doublets using a multiplex cytokine assay (V-PLEX Proinflammatory Panel 1 (human), MSD Rockville, USA).

Measurements of P-sCD163 were performed in doublets by a validated in-house sandwich enzyme-linked immunosorbent assays in plasma using a BEP-2000 ELISA-analyser (Dade Behring), as previously described; sCD163 is stable for years at − 80 °C and when exposed to repeated freeze and thaw cycles [17]. Control samples were included in each run.

### Urinary samples

The patients collected urine for 24 h at home or at the department in a designated container for analysis of copper and zinc excretion.

### Galactose eliminations capacity (GEC)

Only the liver eliminates galactose and the maximum velocity of the elimination quantitates the metabolic liver function [15]. The GEC was measured after a single dose of intravenously injected galactose, followed by analysis of the blood galactose concentration decline over time, essentially as previously described [14]. The results are reported as mmol galactose eliminated per minute (mmol/min) and the patient value as a percentage of normal value (%).

### Fibroscan and ultrasound

Liver fibrosis was estimated by liver stiffness using transient elastography (FibroScan, Echosens, Paris, France) [18]. This was supplemented by a specialist ultrasound examination of the liver [19].

### Statistical methods

Data are presented with median values and interquartile ranges (IQR). Groups were compared by the Wilcoxon Mann-Whitney test. Spearman’s Rho described non-parametric correlations. We performed a linear regression analysis with the GEC as the dependent variable and sCD163 and Fibroscan values as independent variables. All measurements were normally distributed and used as continuous variables in the model. *P*-values< 0.05 were considered statistically significant.

## Results

### Patients and controls

Of the 29 patients, 14 were women and 15 men, with a median age of 35 years. Time since diagnosis showed large variation ranging from 1 year to 50 years, with a median of 16 years (12–26). Seventeen patients were treated with zink either alone (*n* = 8), or in combination with D-penicillamine (*n* = 6) or with Trientrine dihydrochloride (*n* = 3). Nine patients were treated with D-pencillamine alone and three patients were treated with Trientrine dihydrochloride alone. The patients’ median model of end-stage liver disease (MELD)-score was 8 (7–9) and six patients (21%) presented with a MELD-score ≥ 10. Their median copper excretion was 5.3 μmol/24 h (1.7–8.1) and their median zinc excretion was 37.1 μmol/24 h (16.0–68.3). Further patient data are listed in Table 1.

**Table 1:** Patient characteristics

	Patients	Controls	Normal values
Gender (M/F)	15/14	10/9	
Age (years)	35 (24–50)	44 (38–57)	
BMI	23.0 (20.6–24.1)		
Amylase (U/L)	43 (25–59)		10–65
Lactatdehydrogenase (U/L)	161 (136–180)		105–205
ALT (U/L)	39 (24–75)		10–70
Bilirubin (umol/L)	9 (8–14)		5–25
Alkaline phosphatase (U/L)	96 (74–125)		35–105
PP factor 2, 7, 10	0.82 (0.69-0.90)		0.6-1.30
Potassium (mmol/L)	4.0 (3.8–4.1)		3.5–4.6
Sodium (mmol/L)	140 (139–142)		137–145
Albumine (g/L)	38 (36–41)		36–48
Creatinine (umol/L)	69.0 (60.0–87.0)		45–105
eGFR (mL/min 1.73m^2^)	91 (89–91)		> 60
B-leukocytes (10^9/l)	6.1 (5.1–6.5)		3.5–10.0
Haemoglobin (mmol/L)	8.9 (8.2–9.2)		7.3–10.5
Platelets (10^9/L)	204 (151–242)		165–400
Iron (umol/L)	15 (12–21)		9–34
Transferrin (umol/L)	34 (31–37)		24–41
Transferrin-saturation	0.3 (0.2–0.3)		0.10–0.57
Haptoglobin (g/L)	0.9 (0.6–1.3)		0.35–1.85
Zinc in blood (umol/L)	17 (12–25)		10–19
Zinc in 24-h urine (umol/24 h)	37.10 (16.00–68.25)		6–12
Copper in 24-h urine (umol/24 h)	5.3 (1.7–8.1)		0–1.5
MELD-score	8 (7–9)		

Parameters are presented as medians (interquartile range). BMI, body mass index; ALT, alanine transferase; MELD, Model of End-stage Liver Disease

The healthy control persons were 9 females and 10 males and had a median age of 44 years (38–57), slightly older than the patients (*p* = 0.09).

### sCD163 and biochemistry

The median sCD163 concentration was 2.96 mg/L (1.97–3.93) which is high in the reference interval of healthy individuals of 0.69–3.86 mg/L [7]. Seven patients (24%) had a sCD163 concentration above the upper normal limit. The controls had a median sCD163 of about half of that of the patients, 1.51 mg/L (1.31–1.84), *p* < 0.00001).

A high sCD163 tended to correlate with ALT while there was no correlation with albumin, bilirubin, platelets or MELD score (Table 2). However, high sCD163 values correlated significantly with high TNF-α, IL-6 and IL-8. The median BMI was 23.0 (20.6–24.1) and did not correlate with sCD163. sCD163 fell significantly with age (rho = 0.56, *p* < 0.001). The amount of copper and zinc excreted in the 24-h urinary samples did not correlate with sCD163 (copper: *p* = 0.73; zinc: *p* = 0.16).

**Table 2:** Correlations between sCD163 and clinical and biochemical parameters

Parameters	sCD163	
	rho	p-value
Age	**0.56**	**0.00**
BMI	0.02	0.91
ALT	0.34	0.07
Albumin	−0.33	0.08
Bilirubin	−0.04	0.84
Platelets	−0.15	0.43
IL-6	**0.50**	**0.006**
IL-8	**0.62**	**< 0.001**
TNF-α	**0.54**	**0.002**
GEC	**−0.51**	**0.02**
Liver stiffness	0.14	0.46
MELD	0.27	0.16

*BMI* body mass index, *ALT* alanine transferase, *IL* interleukin, *TNF-α* tumor necrosis factor-α, *GEC* galactose elimination capacity, *MELD* Model of End-stage Liver Disease. Values in bold indicate significance below 0.05

### Galactose elimination capacity (GEC)

**Fig. 2:**
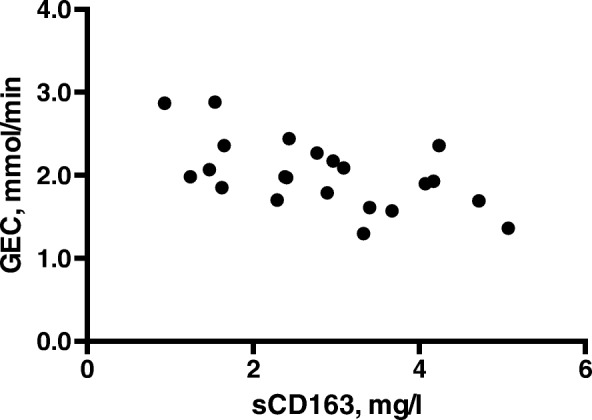
The correlation between sCD163 levels and the galactose elimination capacity (GEC) in patients with WD

The patients’ median GEC was 1.98 mmol/min (1.70–2.30), which on average corresponds to 73% (60–85) of their expected metabolic liver function. Nine patients (31%) had a GEC ≤ 65% of expected liver function. High sCD163 values correlated significantly with low GEC mmol/min-values (rho = − 0.51, *p* = 0.02) (Fig. 2) and with the GEC patient/normal-values, (rho = − 0.47, *p* = 0.03).

### Fibroscan and ultrasound

The median Fibroscan stiffness was 6.7 kPa (5.3–9.5). Thirteen patients (45%) had a value below 7 kPa, the accepted threshold for fibrosis. There was no correlation between the sCD163 and the Fibroscan measures (rho = 0.14, *p* = 0.46).

The ultrasound examination was normal in 7 patients, showed inhomogeneous liver in 6 patients, steatosis in 9 patients, fibrosis in one patient, and cirrhosis in 3 patients. The median sCD163 in the patients with cirrhosis was 3.40 (3.33–5.07) and similar to that in the others, 2.96 (2.29–4.07) mg/l (*p* = 0.21).

There was a tendency for liver stiffness to negatively correlate with the patient GEC/normal (rho = − 0.40, *p* = 0.07), but the correlations were not significant, neither when using the exact values in mmol/min (rho = − 0.28, p = 0.21) or the corrected values in percent (patient/normal).

In a linear regression model, low GEC levels are associated with higher sCD163 levels (*p* = 0.01) independently of liver stiffness (*p* = 0.65).

## Discussion

This is the first description of inflammatory Kupffer cell activation in WD patients, as estimated from their sCD163 values. The central findings were that the patients did show varying signs of macrophage activation, and that the degree of the activation was closely related to the patients’ loss of metabolic liver function measured by the GEC.

The main strength is the inclusion of practically the entire Danish Wilson cohort and the use of well-evaluated and validated methods. The cross-sectional design of the study, however, holds limitations. It was only possible for us to measure sCD163 at a certain time point, which is not well defined in terms of e.g. disease duration, current treatment, or treatment duration. Further, other (even temporary) comorbid inflammatory conditions may affect the sCD163, although no patient was suspected of such problems. Of note, our cohort was clinically and therapeutically stable so the findings we present are likely conservative compared to patients with more active WD. This line should be pursued in future research.

sCD163 has previously been measured in several other and more prevalent liver diseases. The highest values were in patients with acute liver failure [20], followed by acute-on-chronic liver failure [13], alcoholic hepatitis, alcoholic cirrhosis, autoimmune hepatitis, and chronic viral hepatitis [5, 21, 22]. The sCD163 values of our patients were in the moderate range found in patients with early fibrosis from hepatitis B and C and in non-alcoholic fatty liver disease (NAFLD) [6, 12], but were still higher than in healthy persons. Gender distribution was similar in both groups and so does not explain the difference in sCD163 (21).

WD is characterized by hepatocyte cytoplasmic copper accumulation [23], which by oxidative stress may lead to hepatocyte injury and -death [24, 25]. The role of activated Kupffer cells, such as we measure by the sCD63, is to produce cytokines and growth factors, which maintain B-lymphocyte activation and induce stellate cells to become myofibroblasts [26] that produce collagen. This chain of events is in accordance with the association we found between sCD163 and the pro-inflammatory cytokines TNF-α and Il-6. Further, TNF-α and IL-6 are released by Kupffer cells in the inflammatory cascades and may thereby promote liver damage [27]. We expected an association between sCD163 and liver stiffness, as these phenomena are associated in e.g. chronic viral hepatitis [28]. However, we did not find such an association and neither could we show higher levels in the patients with signs of cirrhosis on ultrasound. The most likely explanation is the stability of the patients resulting in a relatively low level of sCD163, combined with the low sample size of 29 patients, which may raise questions regarding sample size and risks of a type 2 error.

Systematic use of measures of liver disease severity is sparse in WD patients, and even more so quantitative measurements of metabolic liver function. We used the GEC for that purpose but we have no other report to compare with. The GEC is a measure of remnant metabolic liver function and not a diagnostic test for cirrhosis. Patients with stable, ‘inactive’ cirrhosis and no ongoing liver injury may have a normal GEC and a good prognosis. Patients without chronic liver disease but prominent liver inflammation may have a low GEC and a poor prognosis (e.g. alcoholic hepatitis or autoimmune hepatitis). Thus, it is quite conceivable that liver inflammation plays a major role for the metabolic liver function. In patients diagnosed with cirrhosis of different aetiologies, a decrease in GEC is associated with a higher mortality [14], as in patients with fulminant liver failure [29]. Inflammation and fibrosis may affect the GEC [15]. The GEC level, we observe, corresponds to a moderately decreased but relatively well-preserved liver function; however, even moderate reductions in GEC are associated with worsened long-term liver-related prognosis [14, 30]. In the present study, we showed a negative correlation between sCD163 and GEC (Fig. 2), which is in accordance with previous studies in patients with cirrhosis [8, 10]. The decrease in metabolic liver function did not seem to be dependent on the liver stiffness. Our findings suggest that in WD inflammatory Kupffer cell activation may be involved in the loss of liver function and that measurement of the sCD163 results in information that is not obtained by fibroscans. Thus, sCD163 may serve as a non-invasive biomarker of loss of liver function in WD which fibroscans evidently cannot.

## Conclusions

In conclusion, sCD163 levels were higher in WD patients compared to healthy controls and 24% showed levels above the upper normal range. Further, Kupffer cell activation assessed by the sCD163 was associated with the loss of metabolic liver function, which did not seem to be related to the degree of fibrosis. The findings suggest that copper mediated Kupffer cell activation may be involved in the loss of liver function in WD.

## Abbreviations

ALT: Alaninaminotransaminase
GEC: Galactose elimination capacity
IL-6: Interleukin-6
IL-8: Interleukin-8
MMPs: Matrix metalloproteinase
PBMCs: Peripheral blood mononuclear cells
sCD163: Soluble CD163
TIMPs: Tissue inhibitors of metalloproteinase
TNF-α: Tumor necrosis factor-α
WD: Wilsons disease
